# Applying Learning Analytics to Detect Sequences of Actions and Common Errors in a Geometry Game †

**DOI:** 10.3390/s21041025

**Published:** 2021-02-03

**Authors:** Manuel J. Gomez, José A. Ruipérez-Valiente, Pedro A. Martínez, Yoon Jeon Kim

**Affiliations:** 1 Faculty of Computer Science, University of Murcia, 30008 Murcia, Spain; manueljesus.gomezm@um.es (M.J.G.); pedroantonio.martinezs@um.es (P.A.M.); 2 Playful Journey Lab, Massachusetts Institute of Technology, Cambridge, MA 02139, USA; yjk7@mit.edu

**Keywords:** educational games, learning analytics, game-based assessment, sequence mining, visualization dashboard

## Abstract

Games have become one of the most popular activities across cultures and ages. There is ample evidence that supports the benefits of using games for learning and assessment. However, incorporating game activities as part of the curriculum in schools remains limited. Some of the barriers for broader adoption in classrooms is the lack of actionable assessment data, the fact that teachers often do not have a clear sense of how students are interacting with the game, and it is unclear if the gameplay is leading to productive learning. To address this gap, we seek to provide sequence and process mining metrics to teachers that are easily interpretable and actionable. More specifically, we build our work on top of *Shadowspect*, a three-dimensional geometry game that has been developed to measure geometry skills as well other cognitive and noncognitive skills. We use data from its implementation across schools in the U.S. to implement two sequence and process mining metrics in an interactive dashboard for teachers. The final objective is to facilitate that teachers can understand the sequence of actions and common errors of students using *Shadowspect* so they can better understand the process, make proper assessment, and conduct personalized interventions when appropriate.

## 1. Introduction

Digital games have become a significant part of families and, specifically, young people around the world. According to a survey conducted in 2020 in the U.S. [1], nearly three-quarters (73%) of American parents believe that video games can be educational for their children, more than half of parents (55%) say they play games with their children, and 92% pay attention to the games their children play. During the COVID-19 pandemic, we have also seen the benefits of games to help people cope with stressful situations, as well as keeping people socially connected around the world while staying at home. Moreover, during the last decade there has been a growing interest in using games in educational settings, not simply because “it is what kids are paying attention to”, but because well-designed games are closely aligned with the design of good educational experiences [2,3].

In the past 10 years, numerous studies (see the work of Clark et al. [4] for a meta-analysis) have reported that games can be more effective than other traditional teaching methods to support both content learning and skill development. Additionally, many studies report teachers’ positive perceptions regarding the use of games in classrooms to promote students’ engagement, motivation, and learning [5]. Despite the benefits of educational games as well as teachers’ positive perceptions, the actual implementation of game-based curriculum in schools still remains rather limited. A 2014 survey [6] reported that 57% of the middle school teachers respondents use games weekly or more often in their teaching, and the majority are at least moderately comfortable using games as a teaching tool. However, they also found that 33% of the respondents were unsure about how to integrate game activities with the regular curriculum. Therefore, it is crucial to provide guidelines that can facilitate teachers to deploy games in the classroom a easier and more flexible way.

One of the predominant challenges that teachers face when they use games in their classrooms is that they cannot have a clear sense of how students are progressing, which students are confused and why, or who is experiencing unproductive struggle within the game. That is, unlike nondigital curricular materials such as worksheets that can be easily reviewed, teachers cannot easily identify different students’ needs by just hovering over their gameplay. Interpreting the raw data generated from the gameplay requires a high level of data literacy skills to understand the reliability and origin of the data [7], but teachers often do not have sufficient training and familiarity to deal with this issue. Providing interactive and intuitive data visualization tools can help teachers to support better integration of digital games with regular curriculum. In that way, teachers can make meaningful instructional decisions by consuming these metrics and visualizations.

Due the highly interactive and open-ended nature of game environments, they present rich opportunities to assess how students are learning based on the process instead of simply relying on the final game performance [8,9,10]. For example, Harpstead et al. [11] discuss how the open-ended nature of the game environment can lead to development conceptual knowledge as well as learning through failure. However, these open-ended environments also present some challenges. An open issue that we usually find in games for learning is to know and understand players’ actions within the game. Therefore, we can benefit from the application of learning analytics techniques to make a meaningful use of the complex and vast data generated from gameplay [10,12].

In this paper, we report two sequence and process mining metrics that we believe are actionable and interpretable by teachers: sequences of actions within puzzles and common errors related to the puzzle solution. We use *Shadowspect*, which is a 3D geometry computer game where students can create geometric shapes such as cones or spheres to solve 3D puzzles, developing their geometric, dimensional, and spatial reasoning skills. When students use *Shadowspect* their interaction with the game environment is collected in the form of large amounts of clickstream data, thus that data can be analyzed and transformed into useful information. In that way, teachers can better understand students’ interaction with the game and analyze their performance, allowing them to make instructional decisions quickly. More specifically, this paper addresses the following objectives:

1. To propose two sequence and process mining metrics: one to analyze the sequences of actions performed by students and another one to analyze their most common errors by puzzle.
2. To develop a set of visualizations embedded in an interactive dashboard that allows teachers to monitor students’ interaction with the game in real time.
3. To exemplify the potential of these metrics and visualizations with two uses cases from data collected in K12 schools across the US using *Shadowspect*.

The rest of the paper is organized as follows. Section 2 reviews background literature on educational games, sequence and process mining, learning analytics, and the development of visualization dashboards in educational environments. Section 3 describes the methods, including the general process of the research, and an overview of *Shadowspect* as well as the data collection. Section 4 presents the definition of the two metrics. Section 5 describes the visualization design and a brief presentation of our dashboard. Next, Section 6 describes two uses cases using the metrics and visualizations developed. Then, we finalize the paper with discussion in Section 7 and conclusions and future work in Section 8.

## 2. Related Work

In this section we present a review of the literature in the three areas that are most related to our work: in Section 2.1 we present literature related to games in education, in Section 2.2 we review sequence and process mining studies, and finally in Section 2.3 we present some works that have been developed in the area of visualizations and dashboards to support learning.

### 2.1. Educational Games

As playing games has become a essential as part of young people’s lives around the world, the use of games for formal education has become widely accepted. There are several reasons that drive the interest of using games in school education. First, in comparison to traditional teaching methods, games are more engaging and fun, and at the same time allow students to hone their skills and build knowledge while they play [13]. This can be attractive to teachers since students frequently find textbooks less appealing and overcrowded [14]. Second, teachers who are often required to teach to learners at multiple levels find it difficult to deliver all the materials. Games can be flexible in that they can be consumed by students autonomously at home or in class, facilitating instructors to better distribute their efforts. Third, due to the open-ended nature of game environments, games present a new opportunity for students to learn from experimenting and exploring authentic and complex problems. The information obtained from the gameplay can be used not only to help teachers manage their classes, understand their students’ learning processes, and reflect on their own teaching methods but also to support learners’ self-awareness of their own actions and to provide them with personalized feedback [15].

However, educators need to be cautious about believing that all games are equally effective in teaching and learning. Whatever the goal they might have, for games and simulations to be successful, they need structural elements to give them shape, and this often comes from the rules of gameplay and/or digital enhancements [16]. The biggest strength of games is that they create learning opportunities and experiences that might otherwise never be able to be created in the traditional classroom [17], yet students still need guided facilitation from the teacher to make games instructionally relevant in the classroom. In addition, many of the existing games for learning are far from having tapped their full pedagogical design potential. For example, when compared to commercially successful entertainment games such as Grand Theft Auto 3 [18], digital games for learning are not as engaging or immersive. Therefore, it is critical to have a design that can be equally fun and instructive, to fully promote challenges, co-operation, engagement, and the development of problem-solving strategies [19].

As a result of this growing use of technology, nowadays we can obtain data from almost everywhere. The increasing availability of educational data provides the educational researcher with numerous opportunities to use analytics to extract useful knowledge to enhance teaching and learning [20]. A game (educational or not) can generate vast amounts of interaction data, even in a short game-play session. The application of data mining and visualization techniques on player interaction logs can provide very valuable insights to different stakeholders regarding how players are interacting with the game [21]. However, the difficulty of measuring learning outcomes achieved through educational games have been one of the main barriers for successful deployment and adoption of educational games within formal education [22].

### 2.2. Sequence and Process Mining

Learning analytics has been defined as the “measurement, collection, analysis and reporting of data about learners and their contexts, for purposes of understanding and optimizing learning and the environments in which it occurs” [23], and sequence and process mining are two of the most prominent techniques applied within this area. Although sequence and process mining are two related areas from a technical viewpoint, conceptually, their objectives are not exactly the same. The objective in process mining is to discover underlying processes from data. The main difference is that process mining is defined from the perspective of an application, while sequence mining is more general and provides approaches for analyzing any types of sequences. Unlike sequence mining, process mining is defined for a specific type of data, like business or other types of processes. In sequence mining, the objective is to find common patterns between data examples where the values are delivered in a sequence [24]. As business logs are also sequences of events, sequence mining can also be applied to these logs. We find in those cases an overlap between these two areas. Analyzing sequences allow us to examine the specific processes students perform while interacting with a learning environment and not just outcome performance measures [25].

Sequence mining is becoming increasingly valuable to understand how students learn in technology-enhanced learning environments, particularly to examine whether their learning process was productive or not [26]. The key concept is to extract meaningful knowledge about students’ learning process, in order to provide insightful information to the teachers to enable interventions [27]. For example, Taub et al. [26] found that there were different types of participants who played their game, allowing teachers to identify each participants’ behavior and make decisions in consequence.

Despite several studies having revealed the effectiveness of game-based assessment for learning, few studies have aimed to use sequence mining to investigate the effectiveness of educational games in learning [26]. As an example, Kinnebrew and Biswas [28] studied the transformation of sequences of events using action features, such as activity categorizations, relevance and timing between actions, and the repetition of analogous actions. Martinez et al. [29] developed a data and sequence mining approach that consisted of mining both the raw human computer interactions and the compact logged actions, clustering similar frequent patterns based on edit distance and analyzing the proportion of these clusters. However, this approach can also result in a very large number of frequent patterns That is why a major challenge in the analysis of frequent patterns is limiting the large sets of results to those patterns that are the real important ones [30].

As raw data can contain a large amount of information which is not related to the students’ interaction, it is very important to transform these data into a clean and actionable sequence of actions. While these sequence mining techniques can be used across different contexts, in this research we apply them on educational data to examine the specific processes students follow during the learning process [30]. We apply sequence mining to analyze students’ sequences of actions while playing the different puzzles included in the game, allowing teachers to monitor each student’s behavior while playing the game as well as the common errors made in the solving process. Besides process and sequence mining, stream data mining [31] and knowledge tracing have been used in other studies to track and evaluate students’ behavior over time. Specifically, in knowledge tracing, the cognitive model is used to interpret each student’s action and follow the student’s step-by-step path through the problem space [32].

### 2.3. Visualization Dashboards

Once the information is obtained after analyzing the data, the next step is to present it in an easily and actionable way. This will help people involved in the learning process (e.g., teachers, students, or other stakeholders) to understand the information provided. Visualizations are one of the most important components of research presentation and communication, because of their ability to represent large amounts of data [33] and also because it is easier for the brain to comprehend an image versus words or numbers [34]. In this research, we use visualizations to graphically represent the information we have obtained from our sequence mining metrics. When creating visualizations, it is important to follow the guideline proposed in [35]: create the simplest graph that conveys the information you want to convey. A large variety of visualizations can be used in data mining [36] including 2D and 3D scatter plots, heat maps, polar charts, or contour plots, among many others. Another key aspect is developing the dashboard with an architecture than can scale and be deployed properly within the educational context [37].

These visualizations should be easily accessible through an interactive interface to be used by nontechnical users, which is commonly known as a dashboard. We can define a dashboard as a visual display of the most important information needed to achieve one or more objectives that has been consolidated on a single computer screen so it can be monitored at a glance [38]. There are four essential characteristics that every dashboard should meet: (1) dashboards are visual displays, (2) dashboards display the information needed to achieve specific objectives, (3) a dashboard fits on a single computer screen, and (4) dashboards are used to monitor information at a glance. In recent years, several dashboard applications have been developed to support learning and teaching. Most of these dashboards are deployed to support teachers to gain a better overview of course activity, to reflect on their teaching practice, and to find students at risk or that are struggling with certain contents [39]. However, most of these dashboards do not address the final application, which is making decisions based on the feedback provided by the metrics available in the dashboard.

Previous studies have developed dashboards and visualizations successfully in other types of learning environments. For example, in the area of games for learning, Martínez et al. [40], Ruiperez-Valiente et al. [41] have developed visualizations to simply represent data from students’ interaction with games. Others researchers have implemented dashboards in different educational contexts, such as massive open online courses [42] or intelligent tutoring systems [43]. Other authors, like Verbert et al. [44], have developed dashboards that allow students and teachers to visualize their grades and predict the future marks based on the current ones. Naranjo et al. [45] developed an dashboard that provides the instructor with visualizations that depict the aggregated usage of resources by all the students during a certain time, also allowing the self-regulation of students, as the dashboard also depicts the percentage of progress for each lab session and the pending actions by the student.

In this work we go a step further, by presenting a new approach to visualize sequence mining analyses in an educational game. We developed autogenerated visualizations embedded in a dashboard that uses friendly icons for teachers to easily interpret them without needing a high data and computer science literacy. This provides a more integral and robust solution to support teachers implementing digital games in the classroom.

## 3. Methods

In this section, we introduce an overview of the system, the educational game used, and the context and data collection of this research.

### 3.1. Overview of the System

**Figure 1:**
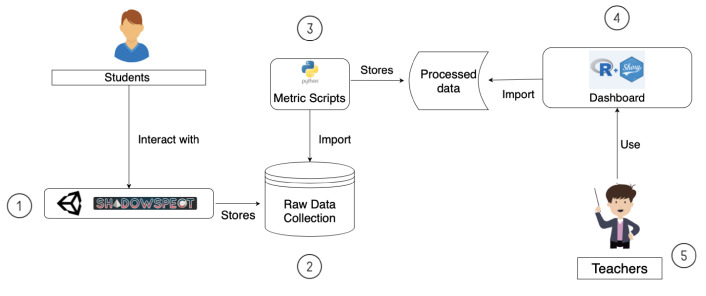
System’s general overview diagram.

In Figure 1 we can see the complete process of our work from the students interacting with the game to teachers using the dashboard developed. Let us see each step in detail:

1. In the first step, students interact with *Shadowspect*. The game has been built using Unity Engine and its deployed as a web application hosted in a web server.
2. The game collects every student’s interaction with the game and stores it in a database.
3. Using the data collection obtained in the second step, metrics are calculated. Each one of the metrics that we have defined is a separate function that computes the required data output as defined in a Python script.
4. The metric’s output is stored as processed data and used by our dashboard. We have developed the dashboard using Shiny’s R framework and we have deployed it on ShinyApps web server. This brings a good number of benefits, such as that the entire deployment pipeline is very easy as it does not need any hardware or configuration of the system.
5. On the last step, we have the teachers, that are using *Shadowspect* in their classes and are the ones that can access the Shiny dashboard environment to visualize what their students are doing.

### 3.2. Shadowspect

**Figure 2:**
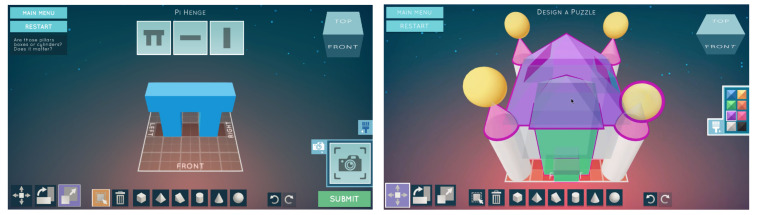
Two gameplay examples in *Shadowspect*.

We use *Shadowspect*, a game-based assessment tool that aims to provide metrics related to geometry content and other behavioral and cognitive constructs. *Shadowspect* has been designed explicitly as a formative assessment tool to measure math content standards (e.g., visualize relationships between 2D and 3D objects), thus teachers can use it in their core math curriculum (see Figure 2).

When students begin a puzzle, they receive a set of silhouettes from different views that represent the figure they need to create, which will be composed of other primitive shapes the student can put into the scenario. The primitive shapes that students can create are cubes, pyramids, ramps, cylinders, cones, and spheres. Depending on the level and difficulty, the puzzle may restrict the quantity or type of shapes they can create. After putting these shapes in the scenario, they can also scale, move, and rotate the shapes in order to build a figure that solves the puzzle. Students can move the camera to see the figure they are building from different perspectives and then use the “Snapshot” functionality to generate the silhouette and see how close they are to the objective. Finally, they can submit the puzzle and the game will evaluate the solution and provide them with feedback (see a demo online [46]).

In the version of *Shadowspect* that we have used in this work, we have 9 tutorial, 9 intermediate, and 12 advanced levels. The tutorial levels aim to teach the basic functionality of the game, so that students can learn how to build different primitives, scale and rotate them, how to change the perspective, take snapshots, and so on. The intermediate levels allow students more freedom so they will not receive so much help to solve puzzles and then the advanced levels pretend to be a real challenge for students who have gained experience with previous levels before. This set of levels provides a linear sequence of increasing difficulty puzzles. However, students can move from any puzzle to another, regardless of its difficulty or order.

### 3.3. Educational Context and Data Collection

The data used for this paper was collected as part of the initial data collection to build the assessment machinery of *Shadowspect*. The team recruited seven teachers who used the game in their theory math classes for at least two hours (class grade from 7th grade to 10th). In this paper, we represent a case scenario of how a teacher could use these visualizations to monitor the progress of their students in her classroom. All student interactions with the game were collected and stored in a MySQL database, and we did not collect any identifiable or personal data from the users except for a nickname provided by themselves. The complete data collection from a total of 322 students includes around 428,000 events (an average of 1320 events per user). Students were active in the game environment for 260 h (an average of 0.82 active hours per student), and students solved a total of 3802 puzzles (an average of 13 puzzles per student). This data collection took place during a semester.

## 4. Sequence and Process Mining Metrics Proposal

In this section, we present our two sequence and process mining metrics: one is related to the sequence of actions within each puzzle attempt and the other is related to common errors in the solving process. The first metric aims to obtain the actions performed by students within a puzzle while playing in order to analyze them and observe potential issues and solutions. The second metric provides a way to automatically identify the most common errors for each puzzle based on the information obtained in the previous metric.

### 4.1. Sequences of Actions

In this metric, the objective is to obtain a sequence of actions for every puzzle attempted by each student, so that we can reconstruct the low-level actions performed while playing *Shadowspect*. This metric is divided into the following two main steps:

1. **Data transformation**: We transform the raw data into an adequate sequence of actions that are representable. This step also includes data cleaning to keep only useful events, in this case we only keep those events related to the puzzle solving process: starting a puzzle, manipulation events (create, delete, scale, rotate or move a shape), snapshots, perspective changes, and puzzles checks.
2. **Data compacting**: We reduce the number of events without compromising the information that is needed for building a sequence of actions. We compact those events that are the same by adding an additional field that indicates the number of times that an event has been performed in a row. For example, if the student has changed the perspective of the game three times in a row, the original data containing three different events will be transformed in a single perspective change event that has been performed three times. When the event is related to the manipulation of shapes, we only compact them if they are related to the same shape identifier.

Applying this algorithm significantly reduced the amount of actions: from 428,000 original actions in the data collection to 107,000 actions (i.e., 75% less events). This is important because this way we lower down the amount of events that need to be interpreted by the teachers. With this metric we obtain a detailed sequence of the actions each student conducted while trying to solve a puzzle, and at the same time we reduce the amount of data making it easier to understand.

### 4.2. Common Errors

This metric uses the output obtained from the sequences of actions and provides a way to identify common errors in the resolution of *Shadowspect* puzzles. Therefore, this allows teachers to quickly identify the errors committed by the students and focus on clarifying those aspects to improve the learning process.

One initial detail to explain is that each puzzle might have multiple solutions. The autosolver algorithm implemented in the game retrieves the silhouettes of the “master” solution and checks if the silhouettes of the current figure submitted by the student match the master solution. Hence, there might be multiple figures that have the same silhouettes as the master solution; however, this is not extremely common. For example, in ten of the puzzles, 80% of the solutions submitted were identical to the the master solution. As the solutions can be different, it is more difficult to find what is an actual error instead of a student trying to solve the puzzle in an alternative way. To solve this issue, we have to make an assumption: we will analyze only puzzles solved using the same shapes of the master solution. In the first place, we apply the sequences of actions metric to obtain the initial input that this metric uses.

Then, we apply an algorithm that has the following two steps:

1. **Identify meaningful events**: We identify the changes a student has made in the shapes between a failed submission and a correct submission. For example, if a student submits a puzzle and the solution is incorrect, and then the student creates a pyramid and deletes a cone in the scenario, those edits are registered by our algorithm as changes between submits.
2. **Compute most common errors**: Once we have registered all those changes after wrong submissions, we group them by puzzle to obtain the shapes and manipulation events that the students have had problems with in each puzzle.

This metric can greatly facilitate error finding for teachers implementing *Shadowspect* in a class.

## 5. Visualization and Dashboard Design

### 5.1. Visualization Design

**Figure 3:**
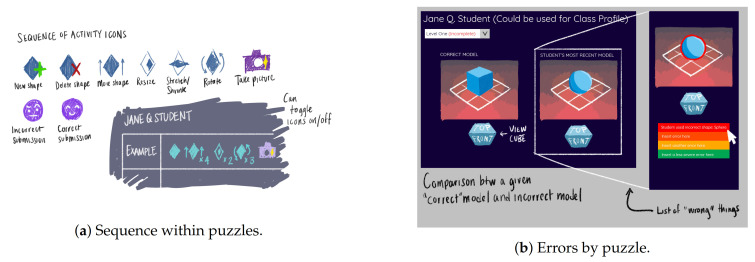
Two examples of digital paper prototype visualizations.

The process to design and develop the final visualizations is iterative based on the feedback and on the interaction between the members of the team. We developed a set of visualization digital paper prototypes that could provide visualization design ideas to be built in the dashboard. For example, Figure 3a represents the digital paper prototype for the sequences of actions metric originally designed by the team. It is very similar to the final visualization that we will present in this paper as part of the dashboard, with icons representing each shape and action. Another example is available in Figure 3b, where we see different ideas of the common errors metric. We see the proposal of detecting incorrect shapes by comparing an incorrect model with the correct one, that is the same idea that we use in our common errors metric.

**Figure 4:**
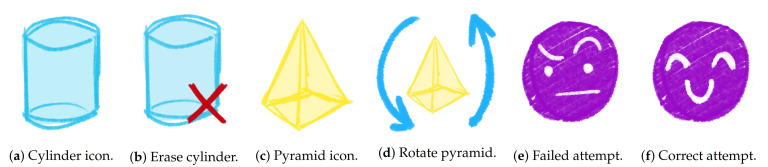
Examples of icons developed.

Moreover, in order to generate representative visualizations, we have also developed some icons that match the different shapes and manipulation events that a student can make through the game. In Figure 4 we can see some examples of the primitive images (Figure 4a,c) that have been used to create composite images taking into account the possible actions that can be made with each shape. For example, in Figure 4b we can see how the deletion of a cylinder is represented. Another example is presented in Figure 4d, with the icon exemplifying the rotation of a pyramid. The last two icons that we introduce here represent an incorrect submission of a puzzle (Figure 4e) and a correct one (Figure 4f).

### 5.2. Dashboard Overview

As we introduced previously, we have developed the dashboard using Shiny’s R framework. That way, we do not need any specific hardware or to configure any part of the system. ShinyApps is also secure-by-design with each application using its own protected environment and access is always encrypted using Secure Sockets Layer (SSL). Finally, the resources allocated to the dashboard are escalable and we do not need to worry about balancing backend resources based on the current workload of the system.

**Figure 5:**
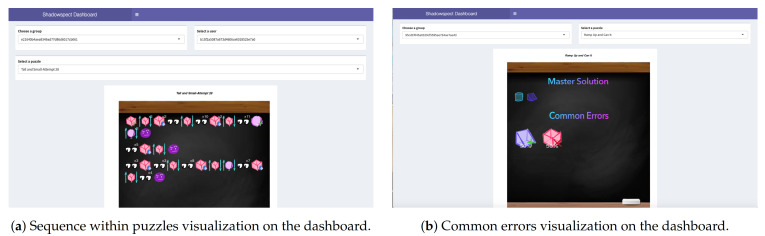
Two examples of our Shiny dashboard interface.

To design the dashboard, as many authors note, we followed the principle that “Everything should be made as simple as possible, but not simpler” [35]. As this dashboard will be used by teachers, we want to prioritize making visualizations easily interpretable, so that they can use the information provided effectively. We have developed a visualization for each of the two metrics that were defined as part of the previous steps. In Figure 5 we can see a couple of examples of the dashboard visualizations. As we see on the upper part of the image, the teacher can select the different groups, users, and puzzles available with the selection boxes. That is how a teacher can monitor every single student simply using the dashboard and the selection boxes.

To better understand the visualizations showed, let us explain how they work. In the first visualization (Figure 5a), for every single action a student can make, we have created an image that represents graphically that action. We can see that the first three actions are the creation of a cube and two movements of that cube. To represent the number of times an action has been performed, we indicate the number of times in the upper-right corner. To represent each type of shape a student can interact with, we use different graphics for each type of shape (cones, ramps⋯), and then we clarify the shape identifier in the puzzle with a number in the bottom-center.

Then, in Figure 5b, we use a similar type of visualization to represent the common errors. This visualization has two parts: on the top, we have the shapes composing the master solution; then, on the bottom, we have the most common errors in that concrete classroom and puzzle, and the percentage over the total number of errors made. In this concrete example, we see that the master solution has a cylinder and a ramp, and the common errors are related with the addition of ramps (50%) and the deletion of cubes (50%). Based on this, as the master solution has a ramp and then a common error is related with the deletion of cubes, we could think that one of the problems that students are having is related to confusing the perspectives of ramps and cubes.

## 6. Uses Cases

This section presents two uses cases of two different puzzles that exemplify how teachers can visualize these metrics to assess different situations. First, we have a use case for “45-Degree Rotations” puzzle, and then another one for “Sugar Cones” puzzle.

### 6.1. “45-Degree Rotations” Puzzle

**Figure 6:**
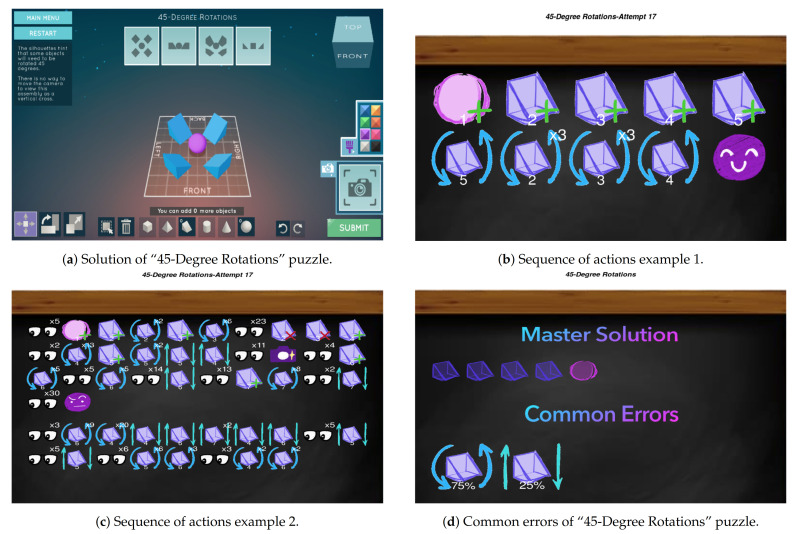
“45-Degree Rotations” puzzle and visualization examples in *Shadowspect*.

In this subsection, we are going to see how two different students solve the same puzzle with different sequences of actions, and then the common errors in the same puzzle for the entire class. First of all, let us introduce “45-Degree Rotations”, which is the puzzle that we are going to use in this subsection. In Figure 6a we see an example of how we can solve this puzzle in *Shadowspect*. As we can observe, the puzzle can be solved using a sphere and four ramps. Let us now see two students’ sequences solving this puzzle.

In Figure 6b we can see that the student has solved the puzzle with a few events. The student creates the sphere and the four ramps, and then rotates the four ramps. Then, the student submits the puzzle and completes it. That way, the student solves the puzzle without making any incorrect submissions, showing confidence on its actions.

In Figure 6c we now see a student solving the same puzzle but with a different sequence of actions. The first thing that we notice is that the student has performed a large amount of actions and an incorrect submit. In addition, after making the incorrect submit, the student keeps trying (changing the perspective, rotating, and moving the ramps...), but eventually the student gives up and leaves the puzzle without solving it correctly.

With these two visualizations, the teacher can monitor each of the two students and then make decisions based on the performance showed by them. That way, the teacher can see that the first student showed in Figure 6b has solved the puzzle without any problem, and the second student shown in Figure 6c has not solved the puzzle and has had difficulties in the solving process. Now, the teacher knows the specific problem of this student and can act accordingly.

After seeing this sequence of actions related to the puzzle, a teacher may want to know if more students have experienced the same problem than the second student. To do that, the teacher can use the common errors metric and visualization to easily detect the most common incorrect actions made by the entire classroom. In Figure 6d, we have an example of the common errors visualization for the same puzzle showed in the sequences of actions visualization. In this specific group, we see that the most common errors are related to the movement (25%) and rotation (75%) of ramps. This matches perfectly with the sequence we have analyzed for the previous student, where, before quitting the puzzle, the student had problems with the rotation and movement of those ramps.

### 6.2. “Sugar Cones” Puzzle

**Figure 7:**
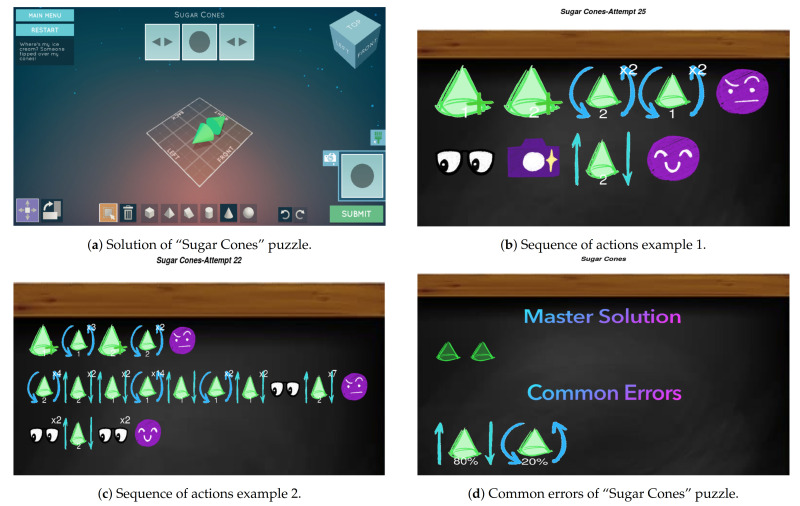
“Sugar Cones” puzzle and visualization examples in *Shadowspect*.

In this subsection we repeat the process but with “Sugar Cones” puzzle. In Figure 7d we see an example of how we can solve this puzzle in *Shadowspect*. As we can observe, the puzzle can be solved using two cones. Let us now see two students’ sequences solving this puzzle.

In Figure 7b we see that this student has solved the puzzle with only nine actions but with an incorrect submit. The student creates the two cones and then rotates them. After seeing that the puzzle submission is incorrect, the student changes the perspective, takes a snapshot and, finally, moves one of the cones, completing the puzzle.

In Figure 7c we now see another student solving “Sugar Cones” but with a different sequence of actions. The student now has two incorrect submits. If we focus on the last line of actions, we see that the student also moves one of the cones before submitting correctly the puzzle, as well as the previous student, and thus it seems that this is a common error in this puzzle.

In Figure 7d we have the common errors visualization for this specific class and puzzle. On the top of the visualization we can see that the master solution has two cones, and on the bottom we see the common errors of the class. As we could expect, the common errors are related to the movement of cones (80%) but also to the rotation (20%), which also matches with the visualization showed in Figure 7c, where the student rotates and moves the cones after several mistakes.

## 7. Discussion

At the beginning of this research we defined the context and main objectives of our work. The two use cases have exemplified the potential of these metrics and visualizations to facilitate that teachers can easily understand and quickly locate the problems of a specific classroom, make adequate decisions, and help their students when appropriate.

With this line of research, we have explored the potential of using temporal sequences to analyze student’s interaction, instead of only using static outputs after playing the game. Our objective was to analyze students’ behavior over time. Other researchers have used sequence and process mining previously to analyze student’s behavior in games. For example, Taub et al. [26] used sequence mining to assess how metacognitive monitoring and scientific reasoning impacted the efficiency of game completion during learning using a game-based learning environment. We can see other examples that also have used process mining, as in [47], where authors use process and sequence mining with predictive purposes. However, our approach is very teacher-centered, clearly targeting the application of sequences within a classroom.

Our use cases support that teachers can use them to analyze students’ sequences in their groups and to detect misconceptions and revise them in the class or with individual students. In his paper, Vellido [48] argued that there are still many barriers to overcome before data techniques become mainstream in real applications. One of them is the interpretability and explainability, which must be guaranteed, as the majority of teachers do not have sufficient knowledge to understand the output provided by data algorithms. In our work, we present actionable and interpretable visualizations, so that teachers can understand quickly the information inferred by the metrics.

As it is important to make sure that the visualizations are usable by teachers, we will be working on obtaining evidences of the interpretability of these visualizations and their usefulness in real classrooms, as we do not have a validation case study with teachers yet, which we consider as our main limitation. Other works have analyzed the impact of visualizations in the learning process and evaluated the usability and effectiveness of dashboards [49]. Mazza and Milani [50] revealed that graphical representations might help instructors to identify individuals that need particular attention, to discover patterns and trends in accesses and discussions, and to reflect on their teaching practice. Another noteworthy limitation is regarding the algorithmic design to detect common errors. The issue is that puzzles can be solved using multiple combinations of shapes. Therefore, the algorithm focuses on computing the errors of only those puzzles that were solved using the master solution. This is done to avoid adding noise to the teachers due to including data from puzzle attempts that were solved in an alternative way, since the majority of the puzzles were solved with the master solution. Additionally, this limitation does not represent a problem to reapply our methodology to detect errors in other interactive educational environments where there is a single solution.

Our work has focused on developing metrics and visualizations, but we have not delved into explaining two other key elements of pedagogical learning analytics design [51]. The first one is related to establishing a connection between the learning analytics and the learning activities that will be performed by students. The second one is related to how the users (students or teachers) will use the chosen analytics as part of the learning activities. In our case scenario, teachers will be using the analytics and visualizations while students solve geometry puzzles within *Shadowspect* at home or in class, in order to facilitate intervening when appropriate. Moreover, based on the information provided, teachers can actively influence the game-based learning activities in order to improve students’ learning outcomes [52].

## 8. Conclusions

The objective of this work was threefold: first, to propose two metrics using sequence mining techniques to provide detailed information in order to infer how students interacted with the puzzles and common errors identified in submitted puzzle solutions. Second, to achieve simple but detailed visualizations for these two metrics, so teachers can monitor students and assess the students’ performance or detect common errors quickly and effectively. Third, to implement a dashboard with these visualizations that can allow teachers to track every single student in their class and present two uses cases. This approach can help alleviate one of the main barriers for implementing educational games in the classroom—difficulty associated with understanding how well or poorly students interact with the game, and locating the problems that the students are having while playing. This also presents an opportunity for educators to provide personalized feedback to their students and better facilitate learning during the game implementation.

As a part of our future work, we will be developing new metrics to continue expanding the dashboard and its possibilities. More nuanced metrics and visualizations will allow students to visualize their mistakes and areas of improvement to better self-regulate their learning process and gain self-awareness of their own activity [53]. We plan to collect additional data from students and teachers via questionnaires in order to more effectively evaluate the impact of the game and visualizations. In addition, we will be working on obtaining evidences of the interpretability of these visualizations and to make them explainable so that teachers can easily intervene. In this way, we can use *Shadowspect* as a robust learning tool with that can be easily implemented by teachers in the classroom and that emphasizes the formative feedback to the student.

## Data Availability

The data presented in this study are available on request from the corresponding author. The data are not publicly available due to privacy concerns.
